# Progression-Free Survival, Prognostic Factors, and Surgical Outcome of Spheno-Orbital Meningiomas

**DOI:** 10.3389/fonc.2021.672228

**Published:** 2021-06-04

**Authors:** Waseem Masalha, Dieter Henrik Heiland, Christine Steiert, Marie T. Krüger, Daniel Schnell, Christian Scheiwe, Oliver Schnell, Anca-L. Grosu, Jürgen Beck, Jürgen Grauvogel

**Affiliations:** ^1^ Department of Neurosurgery, Medical Center – University of Freiburg, Freiburg, Germany; ^2^ Faculty of Medicine, University of Freiburg, Freiburg, Germany; ^3^ Department of Neurosurgery, Cantonal Hospital St. Gallen, St. Gallen, Switzerland; ^4^ Department of Radiation Oncology, Medical Center – University of Freiburg, Freiburg, Germany; ^5^ German Cancer Consortium (DKTK), Partner Site Freiburg, Freiburg, Germany

**Keywords:** spheno-orbital meningioma, meningioma, surgery, postoperative radiotherapy, progression-free survival

## Abstract

**Objective:**

Spheno-orbital meningiomas (SOM) are rare intracranial tumors that arise at the sphenoid wing. These tumors can invade important neurovascular structures making radical resection difficult, while residual tumors often lead to recurrence. The purpose of this study was to evaluate prognostic factors influencing the recurrence and progression-free survival (PFS) rates of spheno-orbital meningiomas, with a particular focus on the role of surgery and postoperative radiotherapy.

**Methods:**

Between 2000 and March 2020, 65 cases of spheno-orbital meningioma were included, of which 50 cases underwent surgical treatment alone, and 15 cases underwent resection and radiotherapy. A Kaplan-Meier analysis was performed to provide median point estimates and PFS rates; further, Cox regression analysis was used to identify significant factors associated with treatment.

**Results:**

Gross total resection significantly reduced the risk of recurrence (p-value = 0.0062). There was no significant benefit for progression-free survival after postoperative radiotherapy (p-value = 0.42). Additionally, spheno-orbital meningiomas with an invasion of the cavernous sinus and intraconal invasion showed significantly worse PFS compared to other locations (p-value = 0.017).

**Conclusion:**

The maximal safe resection remains the most important prognostic factor associated with lower recurrence rates and longer PFS in patients with spheno-orbital meningioma. The invasion of the cavernous sinus and intraconal invasion was an independent factor associated with worse PFS. Patients with postoperative high-precision radiotherapy did not show significantly better PFS due to the small number of patients.

## Introduction

Meningiomas are the most common benign intracranial tumors. They account for approximately 12-15% of intracranial neoplasm (1). Nearly 18% of these are located on the sphenoid wing (1). Spheno-orbital meningioma (SOM) account for 2-9% of all intracranial meningioma (2). They arise from the sphenoid wing and represent a unique category of invasive tumors characterized by pathological hyperostosis of the sphenoid bone. The invasion of the tumor bone together with its proximity to critical structures within the orbit, optic nerve canal, superior orbital fissure, and cavernous sinus lead to classic presentation of proptosis, deterioration of vision, abnormal eye movement, and headaches (3–6).

The surgical treatment for this type of tumor remains controversial. Some authors favor a wait and scan strategy (7). Other authors prefer a complete resection in combination with proptosis correction and visual preservation to achieve a favorable functional outcome (3, 8), despite the fact that gross total resection (Simpson grade I-III) in combination with preserving neurovascular structures is not always achievable. Therefore, this condition remains a surgical challenge with a double purpose of functional preservation and oncological resection (9).

The additional effect of postoperative radiotherapy in meningioma is still a matter of debate. Stereotactic radiotherapy/radiosurgery has been shown to be effective in controlling the growth of meningioma (9, 10), particularly for skull base meningioma (11). However, the literature on spheno-orbital meningioma is very poor regarding postoperative radiotherapy. As yet, the additional benefit of postoperative radiotherapy for spheno-orbital meningiomas is unclear and requires further investigation.

Some authors advocate postoperative radiotherapy, reporting improvement of progression-free survival (12, 13). Bowers and colleagues reported an improvement in proptosis after radiotherapy and no deterioration in visual symptoms (14). Others recommend postoperative radiation treatment only after the progression/recurrence of tumors (15). Others adopt a wait and scan strategy prior to radiotherapy or re-surgery (7, 13).

The purpose of this retrospective study was to investigate the surgical outcome and influence of additional postoperative radiotherapy after surgical resection on progression-free survival in patients with spheno-orbital meningioma. Moreover, we aimed to evaluate prognostic factors that affect the outcome and clinical course of spheno-orbital meningioma.

## Materials and Methods

The present study is a retrospective, single-center study that included patients with SOM who were operated on in our department of neurosurgery between 2000 and 2020.

**Figure 1:**
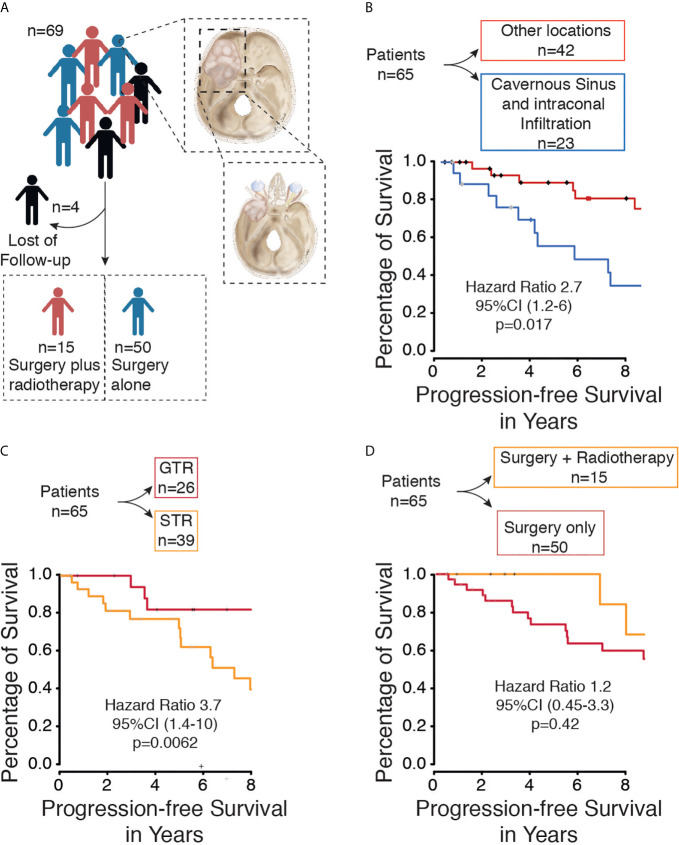
**(A)** Flow diagram of included patients with spheno-orbital meningioma from our database. **(B)** Kaplan-Meier curve for progression-free survival based on tumor location. **(C)** Kaplan-Meier curve for progression-free survival based on extent of resection. **(D)** Kaplan-Meier curve for progression-free survival based on Therapy (Surgery only *vs.* surgery plus radiotherapy).

We only included patients over 18 years of age, histopathological diagnosis of meningioma, and presenting with spheno-orbital meningioma (SOM). SOM was defined as a meningioma involving the sphenoid bone with intraosseous tumor growth that infiltrated the orbit and was associated with a thin “carpet-like” intradural extension. Gender and age of the patients at the time of surgery were recorded. In addition, the symptoms pre- and postoperatively, cranial nerve deficits, tumor extension, extents of resection were assessed according to the Simpson scale. Time of progression/recurrence was defined as soon as tumor progress was identified in follow-up imaging. Simpson’s grade of resection was evaluated based on surgery reports and 3-month follow-up MR and CT imaging. Simpson grades I-III were defined as gross total resection, and Simpson grades IV-V were defined as subtotal resection in our study. The mean follow-up was 10.1 ± 6.4 years. Karnofsky Performance Scale (KPS) and progression-free survival (PFS) were used for oncological and neurological assessment of outcome. New lesions or a growing residual tumor on follow-up MRI scans were defined as tumor recurrence/progression. The first tumors to be diagnosed with defined as primary SOM, whereas tumors were defined as secondary if they were previously treated. Patients with incomplete data were excluded (**Figure 1A**).

### Statistical Analysis

Progression-free survival analyses were conducted using Kaplan-Meier analysis, with the between-group differences analyzed using a log-rank test. Univariate and multivariate Cox regression analyses were also performed. The alpha level was defined as 5%. All statistical analyses were performed using R software (package: survival, ggplot2, MANOVA). Plots were performed by R software package ggplot2. Differences were considered significant at p < 0.05.

## Results

### Patient Data

A total of 69 patients with spheno-orbital meningioma were treated in our neurosurgical department between 2000 and 2020. Four patients were excluded due to a lack of follow-up data (**Figure 1A**). The sex ratio (male/female) was 1:3.3. The surgery group included 50 patients (11 males and 39 females) with a mean age of 56.98 years (standard deviation ±12.52). The surgery plus postoperative radiotherapy group included 15 patients (4 males and 11 females) with a median age of 48.41 years (standard deviation ±10.03) (p=0.0107). Most tumors were WHO grade I (n=50, 77%), but the remaining were WHO grade II (n=15, 23%) (p=1). Frequent symptoms at presentation were headache, exophthalmos, deterioration of vision, and abnormal eye movement. A detailed overview of all parameters is given in **Table 1**.

**Table 1:** Patient data.

Parameter	Surgery N=50	Surgery plus Radiotherapy N=15	
**Age (Years), Mean (SD)**	56.98 ± 12.52	48.41 ± 10.03	p=0.0107*
**Sex (N, %)** **Female** **Male**	39 (78%) 11 (22%)	11 (73%) 4 (27%)	p=0.73**
**Resection grade (N, %)** **GTR** **STR**	24 (31%) 26 (69%)	2 (3%) 13 (97%)	p=0.03***
**Preoperative KPS** **Mean (SD)**	80.2 ± 11.69	82 ± 8.61	p=0.52*
**Postoperative KPS** **Mean (SD)**	80.7 ± 12.69	80.6 ± 7.03	p=0.98*
**WHO** **grade I** **grade II**	40 (80%) 10 (20%)	12 (80%) 3 (20%)	p=1**
**Proliferation index** **<1** **>1**	36 (72%) 14 (28%)	10 (66.6%) 5 (33.3%)	P=0.75**
**Primary** **Secondary**	38 (76%) 12 (24%)	9 (60%) 6 (40%)	p=0.32**

*T-Test.

**Fisher´s exact test.

***Chi-squared test.

KPS, Karnofsky Performance scale.

CI, Confidence interval.

GTR, Gross total resection.

STR, subtotal resection.

SD, Standard-deviation.

### Extent of Tumor Resection

Surgical resection of the tumors was performed on all patients. Gross total resection (Simpson grade I, II, and III) was performed in 26 cases (40%), and subtotal resection (Simpson grade IV and V) was achieved in 39 cases (60%) (**Figure 1C**). Tumor recurrence was found in 4 (6.1%) cases after gross total resection (Simpson grade II, II, and III) and in 17 cases (26.1%) after subtotal resection (Simpson grade IV and V). The performed Kaplan-Meier analysis showed a significantly prolonged PFS in patients with GTR (p = 0.0062) (**Figure 1C**), and the same was seen in the univariate (p ≤ 0.0093) and multivariate analyses (p ≤ 0.0091) (**Table 2**).

**Table 2:** Cox regression analysis.

**Variable clinical and treatment factors**	**Progression-free survival**		**Progression-free survival**	
	HR (95% CI)	p value	HR (95% CI)	p value
	Univariate analysis		Multivariate analysis	
**Sex** **(female *vs* male)**	0.62(0.26-1.4)	0.27		
**Age** **(≥55 *vs* <55)**	0.52(0.21-1.3)	0.14		
**GTR *vs*. STR**	*3.7(1.4-10)*	*0.0093*	*4(1.4-11.12)*	*0.0091*
**Surgery *vs*. surgery plus radiotherapy**	1.2(0.45-3.3)	0.7	2.5(0.86-7.3)	0.09
**Primary *vs* Secondary**	2.1(0.93-4.9)	0.074		
**Preoperative KPS** **(≥80 *vs* <80)**	0.97(0.42-2.2)	0.94		
**Postoperative KPS** **(≥80 *vs* <80)**	0.47(0.19-1.2)	0.11		
**WHO I *vs*. WHO II**	*0.47(1.9-12)*	*0.00076*	*4.3(1.6-11.3)*	*0.0029*
**Proliferation Index (≥1 *vs* <1)**	1.5(0.66-3.6)	0.32		

GTR, Gross total resection.

STR, subtotal resection.

CI, Confidence interval.

HR, Hazard ratio.

KPS, Karnofsky Performance scale.

### Postoperative Radiotherapy

Fractionated high-precision radiotherapy was performed after subtotal resection or in tumors with WHO grade II. Fifteen patients (23%) were treated postoperatively with fractionated high-precision radiotherapy of the residual tumor, of which five patients showed tumor recurrence/progression (7.6%). All tumors were treated with a radiation dose between 50.4 and 54 Gray. In contrast, fifty patients (76.9%) were treated with surgery alone, of which 19 patients (29.2%) showed recurrence/progression of the tumor. The Kaplan-Meier analysis (p=0.42) (**Figure 1D**), as well as the univariate (p=0.7) and multivariate analyses (p=0.09), showed no significant differences between both groups (**Table 2**). However, five years progression-free survival was 0% in patients who were treated postoperatively with radiotherapy and 28% in patients who were treated with surgery only (p=0.0289).

### Surgical Outcome

Surgical morbidities occurred in 10 patients (15.3%), including wound infection (n=4), transient aphasia (n=1), epilepsy (n=3), and CSF fistula (n=1). The mean preoperative Karnofsky Performance Scale (KPS) was 80.61 ± 11.02% (range 30-90%), and the mean postoperative KPS was 80.69 ± 11.58% (range 30-100%). The most affected cranial nerves after surgery were the optic nerve (n=13, 20%), oculomotor nerve (n=9, 13%), abducens nerve (n=10, 15%), and trigeminal nerve (n=10, 15%). Remarkably, permanent deficits of the trigeminal nerve were significantly increased after GTR (p=0.0107) (**Table 3**). A detailed overview of all deficits of the cranial nerves is given in **Table 3**.

**Table 3:** Postoperative cranial nerve deficits.

Cranial Nerve	Postoperative permanent deficits (N, %)	GTRN=26	STRN=39	p-value
I c.n	1(1.5%)	0	1	p=1**
II c.n	11(15%)	4	7	p=0,72**
III c.n	9(13%)	3	6	p=0,73**
IV c.n	4(6%)	0	4	p=0,14**
V c.n	10(15%)	8	2	p=0,0107**
VI c.n	11(15%)	4	7	p=0,72**

c.n, cranial nerve.

GTR, Gross total resection.

STR, subtotal resection.

HR, Hazard ratio.

**Fisher´s exact test.

### Other Factors

Additional univariate and multivariate Cox regression analysis of patients with progressive disease was performed to identify potential prognostic factors for tumor recurrence/progression. The univariate analysis (p = 0.00076) and the multivariate analysis (p = 0.0029) showed an improved PFS in patients with WHO grade I compared to patients with WHO grade II tumors (**Table 2**).

Within the subgroup analysis, a total of 23 patients presented spheno-orbital meningioma with infiltration of the cavernous sinus and intraconal infiltration, of which 13 patients showed tumor recurrence/progression. The Kaplan-Meier analysis (p=0.017) indicated that tumors located in the cavernous sinus or/and intraconal compared with other locations were associated with worse PFS (**Figure 1B**).

## Discussion

This study retrospectively reviewed patients with spheno-orbital meningioma who had received surgery between 2000 and 2020. To our knowledge, this is one of the largest single institutional series focusing on spheno-orbital meningiomas published to date. Our database contains 65 cases of spheno-orbital meningioma, of which 50 were treated with surgery alone, and 15 additionally received stereotactic radiotherapy after surgical resection. The aim of this study was to investigate the role of surgical resection and postoperative radiotherapy after resection to determine possible prognostic factors for tumor recurrence.

### Surgery

It is believed that radical surgery has a positive effect on the PFS and prognosis of patients with all subtypes of meningioma (9). Previous studies reported various gross total resection (GTR) rates in SOM (4, 5, 8, 16, 17). In our study, 40% of cases received GTR, which is in line with other reported series, ranging between 25% and 69% GTR. Further, we were able to confirm that GTR of SOM was associated with significantly better PFS; although, increased neurological impairment of the trigeminal nerve was observed (**Table 3**, p=0.0107). In our study, GTR was defined as Simpson grade I-III, which was achieved in 26 patients, of whom only one patient received a Simpson grade I resection. This patient suffered from severe complications, such as global aphasia and abducens nerve palsy. Simpson grade II was achieved in only two patients, one of whom suffered from a severe lesion of the optic nerve. All other patients received a Simpson grade III resection (n=23).

In our study, STR was achieved in 60% of cases, which is in line with other reports ranging from 31% to 75% (4, 5, 8, 16, 17).

Our results emphasize the common observation in the literature that a radical resection (Simpson grade I and II) of SOM is often impossible without causing severe morbidity (4, 5, 16, 18). Therefore, in our own clinic, the operation aimed at maximal safe resection, paying particular attention to the decompression of the optic nerve canal to maximize visual acuity and the removal of the intraorbital tumor to improve proptosis. Other authors highlighted the importance of symptom-oriented surgery instead of radical surgery (7, 12).

Our results emphasize the importance of a better degree of resection to prolong PFS; however, this should be aimed at while also preserving functionality.

### Radiotherapy

Only a few studies have evaluated the effectiveness of radiotherapy in spheno-orbital meningiomas; accordingly, the treatment strategy varies from institution to institution. In our institution, modern high-precision radiotherapy was performed after STR or in the case of WHO grade II tumors. A total of 15 patients (23%) received postoperative radiotherapy after surgery in this cohort (**Figure 1A**). The five years progression-free survival was significantly (p=0.0289) improved, whereby no significant benefit in the Kaplan-Meier analysis of patients submitted to postoperative radiotherapy due to the small number of patients treated with additional radiotherapy (p=0.42). Additionally, the character of a retrospective study does not allow a bias-free interpretation of these results based on unbalanced follow-up and a selective population that receives radiotherapy containing only STR patients.

In recent years, more and more studies have been published that recommended radiotherapy after subtotal resected meningioma (9–11). However, few reports investigated the efficacy of postoperative radiotherapy for SOMs. Most of the studies reported on small patient cohorts with SOM and postoperative radiotherapy without providing clear conclusions on the role of postoperative radiotherapy (4, 17, 19, 20). Others recommended postoperative radiotherapy in the case of atypical/rapidly progressive tumors (12, 13, 16, 21). Boari and colleagues advised treatment with radiotherapy by the invasion of the superior orbital fissure and cavernous sinus to allow for minimal surgical morbidity (3). We believe that radiotherapy after STR or high-grade meningioma (WHO grade II and III) could prolong PFS. Terpolilli et al. were able to obtain similar results (22). They showed in their retrospective study that early postoperative radiotherapy after STR of orbital-associated meningioma helps to delay tumor recurrence and the need for further treatment while maintaining or even improving visual outcome (22).

### Functional Outcome

One of the goals of the operation is the aesthetic aspect and the reduction of proptosis. This has been achieved in our series by decompression of the orbit, in which tumor-infiltrated parts of the roof and lateral wall of the orbit were resected. In our series, this was achieved in 83% of patients (**Table 4**). Other series reported similar results (7, 13). At the last examination within this study, 79% of patients showed stable and 4% improved visual acuity. The stenosis of the optic canal caused by tumor invasion in SOM leads to a deterioration of visual acuity (23). Unroofing of the optic canal in this series led to stabilization/improvement of visual acuity. Our results are consistent with the reported results of other groups (13, 23). In our cohort, we observed a total of 10 patients with postoperative hypoesthesia in the region of the trigeminal nerve, which was significantly (p=0.0107) associated with GTR (**Table 3**). Other groups observed only sporadic trigeminal nerve deficits and other cranial nerve deficits (5, 8, 12, 14, 19, 24–26). This could be biased by the surgical experience of the surgeon and the aggression of the tumors.

**Table 4:** Visual acuity and proptosis after surgery at last examination.

	Worse (N, %)	Stable (N, %)	Improved (N, %)
**Visual acuity**	11 (17%)	51 (79%)	3 (4%)
**Proptosis**	2 (3%)	9 (14%)	54 (83%)

### Tumor Location and Other Factors

Cavernous sinus invasion and intraconal invasion were associated with significantly (hazard ratio=0.98, p=0.017) worse PFS compared to other locations (**Figure 1B**). The cavernous sinus and the intraconal tumor components were not removed to avoid persistent oculomotor paralysis and venous bleeding, as mentioned by other authors in the past (4, 13, 19). Leroy et al. reported a significant reduction in PFS by an invasion of the cavernous sinus and optic canal (13). However, they found that the optic canal opening was significantly associated with improved PFS. Other groups reported similar results; furthermore, they reported that the invasion of the cavernous sinus and superior orbital fissure was associated with worsening of PFS (4, 13, 19).

### Limitations of the Study

The retrospective nature of this study and the limited external validity within a single institution restricted this study. The small number of patients treated with additional radiotherapy is an additional limitation of this study. Therefore, the statistical power is inadequate in this regard. This limitation may also explain the inability of the study to demonstrate a significant benefit of postoperative radiotherapy in PFS for SOM. Additional limitations imposed by a retrospective study design, such as heterogeneous management strategies, variability in the extent of follow-up, and variability between observers in assessing the extent of resection, must be considered when interpreting the results.

Nonetheless, our study is one of the largest series to date, focusing on the extent of resection of spheno-orbital meningiomas and their postoperative radiotherapy.

## Conclusions

Based on our experience, we summarize our findings:1. Important prognostic factor for determining recurrence was the maximum safe resection with preservation of function. 2. Additional postoperative radiotherapy did not significantly prolong progression-free survival. 3. Tumor invasion into the cavernous sinus and intraconal invasion were independent factors associated with worse PFS. According to our experience and the experience of other groups, the resection of these tumor parts can lead to severe complications. Therefore, postoperative radiotherapy should be considered in such cases.

## Data Availability Statement

The original contributions presented in the study are included in the article/supplementary material. Further inquiries can be directed to the corresponding author.

## Ethics Statement

The studies involving human participants were reviewed and approved by Albert-Ludwigs- Universtität Freiburg Institut für Ethik und Geschichte der Medizin. The ethics committee waived the requirement of written informed consent for participation. All procedures performed in studies involving human participants were in accordance with the ethical standards of the institutional and/or national research committee and with the 1964 Helsinki declaration and its later amendments or comparable ethical standards.

## Author Contributions

WM drafted the manuscript and participated in the data collection. DH participated in the preparation of the manuscript and in the data collection. WM, DH, and MK participated in the design of the figures. OS, JB, DS, CSt and A-LG participated in the preparation of this article by revising it with regard to important intellectual content. CSc and JG coordinated the study and revised the article for important intellectual content. All authors contributed to the article and approved the submitted version.

## Conflict of Interest

The authors declare that the research was conducted in the absence of any commercial or financial relationships that could be construed as a potential conflict of interest.
